# Performance Evaluation of Public Non-Profit Hospitals Using a BP Artificial Neural Network: The Case of Hubei Province in China

**DOI:** 10.3390/ijerph10083619

**Published:** 2013-08-15

**Authors:** Chunhui Li, Chuanhua Yu

**Affiliations:** 1 School of Public Health, Wuhan University, 115 Donghu Road, Wuhan 430071, China; E-Mail: chli0201@hotmail.com; 2 Global Health Institute, Wuhan University, 115 Donghu Road, Wuhan 430071, China

**Keywords:** public non-profit hospitals, health care reform, indicator system, performance evaluation, BP neural network, cross validation

## Abstract

To provide a reference for evaluating public non-profit hospitals in the new environment of medical reform, we established a performance evaluation system for public non-profit hospitals. The new “input-output” performance model for public non-profit hospitals is based on four primary indexes (input, process, output and effect) that include 11 sub-indexes and 41 items. The indicator weights were determined using the analytic hierarchy process (AHP) and entropy weight method. The BP neural network was applied to evaluate the performance of 14 level-3 public non-profit hospitals located in Hubei Province. The most stable BP neural network was produced by comparing different numbers of neurons in the hidden layer and using the “Leave-one-out” Cross Validation method. The performance evaluation system we established for public non-profit hospitals could reflect the basic goal of the new medical health system reform in China. Compared with PLSR, the result indicated that the BP neural network could be used effectively for evaluating the performance public non-profit hospitals.

## 1. Introduction

Since 2009, new health reforms have entered the implementation stage in China. Changes to public non-profit hospitals are an important part of the health reform process. In the past three years, reform has had positive effects, but it has also encountered some problems. 

The only way to evaluate the effects of public non-profit hospital reform and to solve the problems accurately is to establish a performance evaluation system. An evaluation system could help managers make decisions and determine how to improve hospital-performance [1,2,3]. A performance evaluation system for public non-profit hospitals will not only establish a better system for supervising performance but also facilitate evidence-based health policymaking and the regulation of public non-profit hospitals.

In recent years, as public non-profit hospital reform has been implemented, many studies have comprehensively evaluated the operations of domestic public non-profit hospitals. However, the research on the performance of public non-profit hospitals is currently limited to the quality of medical services, service efficiency, service ability and management ability, which are not sufficient to fully evaluate the performance for public non-profit hospitals. Tang *et al.* [4] have used the balanced score card to establish the performance evaluation system for public non-profit hospitals. In Cui *et al.* [5], the evaluation system included investment assets, service quality, financial management and external evaluation. Wang *et al.* [6] have selected several key performance indicators by key success factor. They ignored the embodiment of commonweal in their studies. In addition, the present evaluation system lacks a satisfaction indicator for evaluating medical performance, such as patient satisfaction and medical safety. Thus, the evaluation results will not support the sustainable development of public non-profit hospitals, and they cannot accurately measure the effectiveness of public non-profit hospital reform.

The most important feature of an ideal performance evaluation system is the accuracy of the evaluation results. Thus, it is important to choose reasonable indicators that reflect the purpose of the performance evaluation and to use the proper methods to evaluate performance. 

Recently, the literature on performance evaluations of medical services has dramatically increased in China. Key Performance Indicators (KPIs) have been used widely; the most common indicators are medical costs and medical quality [7]. Subsequently, many methods (including the “Balanced Score Card” (BSC)) have been introduced into the performance evaluations of hospitals [8,9]. Furthermore, fuzzy comprehensive evaluations, fuzzy gray relational analyses and TOPSIS have been applied for evaluating hospitals or making predictions.

However, there are some disadvantages to conducting of fuzzy comprehensive evaluations and fuzzy gray relational analyses. In fuzzy comprehensive evaluations, the weights of the factors are subjective and the membership function is hard to define [10,11]. In addition, because of the narrow theoretical basis of the grey correlation quantitative model, the positive correlation result is contradictory to the actual relationship between the factors [12,13,14]. Considering the questions above, we chose artificial neural networks (ANNs). The neural network more closely represents human thinking. Based on the expert evaluations of the given sample and the knowledge gained from experience, the neural network can be used to compute complex nonlinear relationships, just like human brain [15,16]. Thus, both qualitative analysis and quantitative analysis can be used, and the objectivity of the evaluation results will be ensured. In recent years, artificial neural networks have emerged as tools for clinical decision-making [17], and they may be more successful than traditional statistical models in predicting clinical outcomes [18,19]. ANNs can acquire experiential knowledge expressed through internal connections in a manner similar to the way natural neurons function in the brain, and this knowledge can be made available for use [20]. ANNs, which demonstrate excellent performance in modeling nonlinear relationships that involve a multitude of variables, can potentially be useful tools to alleviate nonlinear problems. However, applications of ANNs in public health monitoring and evaluation are uncommon.

The present evaluation mainly depends on the experience and knowledge of experts, and this information is difficult to express in mathematical formulas. In addition, it is more difficult to accumulate experiences. Artificial neural networks can capture self-study and self-evaluation, and they have high overall collateral and a high capacity for nonlinear relationships. These models can use hidden knowledge expression to integrate knowledge into the interlinked concepts and the linked weights in the network, making it easier to realize the relationship between experience and knowledge [21]. An ANN model with an input layer, an output layer and one or more hidden layers could be an adequate universal approximate of any nonlinear function [22,23]. The input layer comprises the data available for the analysis, and the output layer comprises the outcome (e.g., a prediction, prognosis or evaluation).

Based on the current situation in China and the aim of the performance evaluation, this study used an input-output model to establish proper evaluation indicators. The weighted TOPSIS method and BP artificial neural networks were used to conduct a comprehensive evaluation. We chose 14 level-3 public non-profit hospitals located in Hubei Province for a performance evaluation.

## 2. Methods

### 2.1. Data Collection

Hubei Province lies in central China, and it was one of the first provinces to implement health reform. This evaluation was conducted at large public non-profit hospitals (14 hospitals) in Hubei Province. At the beginning of 2009, the Hubei Health Ministry (HHM) conducted a program to facilitate reforms of public non-profit hospitals. All of the public non-profit hospitals were required to report service and management data to the Hubei Medical Service Information Quality Control Center (HMSIQCC) via Hubei Public Hospital Information Software. The Information Software involves 117 variables, including general information (the number of doctors, beds, *etc.*), hospital management (including training times and infectious disease control), medical service quantity (including information on all services performed in the hospital), medical service quality (including hospitalization outcomes, e.g., diagnosis rates, positive rates, *etc.*), nursing quality control, laboratory quality control, economic efficiency (including income, expenditures, *etc.*), medical safety (including medical negligence and compensation information) and patient satisfaction. The standardized reporting system was developed by HHM, and the doctors and administrators from all of the public non-profit hospitals in Hubei were trained to report the data in a standard format. After the data had been uploaded to the software, the experts in HMSIQCC could exclude abnormal data and check the accuracy of the data, which could then be downloaded for our study. The data used in our study came from the HMSIQCC in the first half of 2012. The integrity and the accuracy of the data were checked by HMSIQCC. 

### 2.2. Establishing the Evaluation System

#### 2.2.1. Indicator Selection

Given our understanding of performance, the input-output model was used as the framework for evaluating public non-profit hospitals. The efficiency and the quality of services hospitals are key concerns for consumers and managers and are widely used in performance evaluations [24,25,26]. Therefore, this study began from these starting points to establish the evaluation indicators.

First, we used experts’ suggestions and the criteria from the literature to select the evaluation indicators. Experts and grassroots workers were invited to complete a questionnaire based on four scientific principles (orientation, comparability, operability and representativeness). After several rounds of expert scoring, we reached a final selection of reasonable, high sensitive, typical indicators that could meet the performance evaluation needs of government managers and public non-profit hospitals.

#### 2.2.2. Tendency Treatment

If a higher value of an indicator indicates better performance, the normalized value can be calculated by Equation (1):

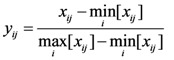
(1)


If a smaller value indicates better performance, the normalized value can be calculated by Equation (2):

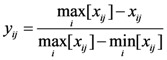
(2)


If a value in a fixed interval indicates better performance, the normalized value can be calculated by Equation (3):

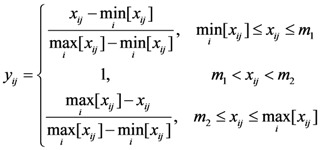
(3)
where *x_ij_* is the value of indicator *j* for the evaluation subject *i*. 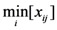 is the minimum value of indicator *j* for all of the evaluation subjects, and 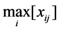 is the maximum value of indicator *j* for all of the evaluation subjects. If the best standard value was not provided, we used *X* ± *S* as the best value (e.g., the daily number of clinic patients for each doctor).

#### 2.2.3. Weight Definition

First, according to the importance of each indicator, we invited the experts to score the indicators, and we then determined level l and 2 index weights using an analytic hierarchy process (AHP). The AHP is a decision-making tool developed by Satty to handle complex, unstructured and multi-factor problems [27]. The AHP involves ranking a set of indices with respect to an overall goal, which is broken down into a set of criteria and indices [28]. The weights of indicators were calculated by conducting pairwise comparisons between the relative importance of the lower evaluation indicator and the relative importance of the upper indicator.

The entropy weight method was used to determine the weights of the level 3 indicators. The weight of indicator *j* can be calculated by Equation (4):

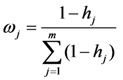
(4)
where entropy *h_j_* as 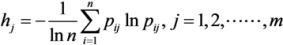. In addition, *p_ij_* ln *p_ij_* is defined as 0 if *p_ij_* = 0. 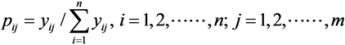.

The comprehensive indicator weights were calculated by determining the product of the weights of the level 1, 2 and 3 evaluation indicators.

### 2.3. Artificial Neural Networks

Artificial neural networks can be defined as a parallel distributed processing method with a large number of processing elements and neurons connected to one another with different connection strengths. The strength of a connection between neurons is the weight. In the beginning of the neural development process, these weights are initially random. They are adjusted in a model calibration phase (called “training”) to minimize the MSE between the calculated outputs and the corresponding target output values for the particular training data set. The testing subset is used to check the performance of the developed network. Various types of ANNs are used for different applications [29].

**Figure 1:**
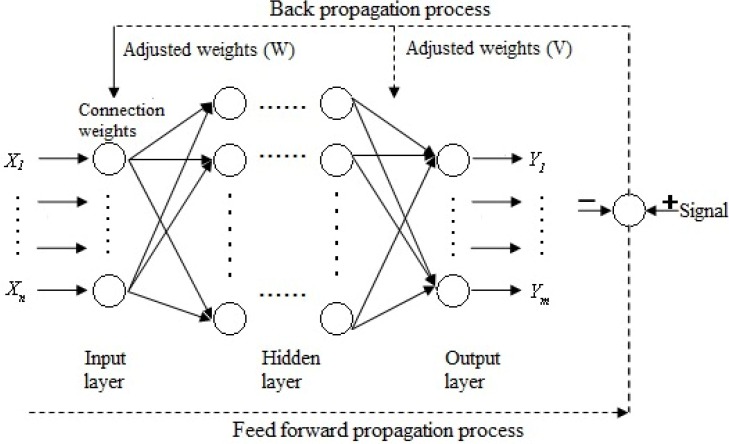
Three-layer BP artificial neural network framework.



BP (back propagation) artificial neural networks (BP-ANN) are typically used for amending errors. BP-ANNs set each quantifiable indicator as the network’s input (*X*) and the result as the output (*Y*)*.* After training enough samples and repeatedly amending the connection weight values (*W*, *V*) and the threshold values among the neurons, the final weight values and threshold values were obtained to indicate correct knowledge (Figure 1).

In the three-layer BP-ANN in our study, the indicators for the performance evaluation system for public non-profit hospitals were used as input variables, and each public non-profit hospital’s performance score as the output. According to the evaluation index system, there were 41 third-level evaluation indicators. The outcome was the performance evaluation; thus, the output layer included only one variable. Therefore, the numbers of neurons in the input and output layers were 41 and 1, respectively. The number of hidden layer neurons can be calculated by Equation (5). The number of neurons in the hidden layer ranged from 8–17.

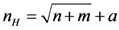
(5)
where *n* is the number of neurons in the input layer, *m* is the number of neurons in the output layer, *a* is the constant, and 1 < *a* < 10.

When BP neural networks are used for public hospital performance evaluation, the data should be normalized before they are trained. In this paper, the MATLAB software normalization function “mapminmax” was adopted, and the normalization interval was 0–1.

The mean squared error (MSE) can be used to determine how well the network output fits the desired output. MSE is defined as follows:

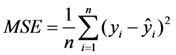
(6)
where *y_i_* is the observed value, *ŷ_i_* is the network output value, and smaller values indicate better performance.

To avoid the effects of using fewer samples for training and to influence the generalization ability of network, the Leave-one-out Cross Validation (LOOCV) method was used to train and test the BP neural network in our study. In LOOCV, if the raw data set has N samples, the model is trained and tested N times. Each time, one sample is selected as the validation sample, and the remaining samples are used as training samples. The cross-validation estimate of the overall accuracy is simply calculated as the average of the N individual accuracy measures. With this method, we attempted to derive reliable results and increase the generalization ability of network [30,31].

The performance of the model can be evaluated using certain statistical indicators, including the coefficient of determination (*R*^2^), the root mean squared error (RMSE) and the mean absolute percentage error (MAPE). These indicators are mathematically defined as follows:

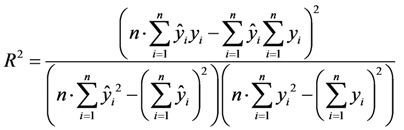
(7)

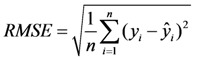
(8)

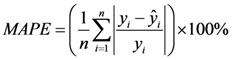
(9)
where *y_i_* is the observed value and *ŷ_i_* is the network output value.

According to Table 1, the most suitable neural network was model with 10 neurons in the hidden layer.

**Table 1:** Statistical indicators for various numbers of neurons in the hidden layer.

Neuron number	*R*^2^	RMSE	MAPE
8	0.9647	0.0229	1.1064
9	0.9505	0.0266	1.4203
10	0.9783	0.0214	1.0858
11	0.9634	0.0258	1.4533
12	0.9753	0.0283	1.1695
13	0.9681	0.0238	0.9469
14	0.9473	0.0271	1.5162
15	0.9629	0.0249	0.9794
16	0.9548	0.0242	1.3341
17	0.9702	0.0221	1.0903

Thus, a 41-10-1 BP neural network was obtained. The learning speed, the maximum numbers of epochs, the target error goal MSE and the minimum performance gradient were set at 0.05, 3,000, 10^−5^, and 10^−5^, respectively. The Levenberg-Marquardt (LM) algorithm was chosen to avoid the time-consuming one-dimension searching [21]. Training stopped when any of these conditions occurred. All of the calculations were performed using MATLAB software (MathWorks, Inc., Natick, MA, USA).

To explain the good performance of BP neural network, we compared BP neural network with partial least-squares regression (PLSR). PLSR is a new multivariate statistical analysis method. The advantages of PLSR are exhibited in dealing with the problems: low sample, more independent variables and multi-correlation [32]. The coefficient of determination (*R*^2^) was used to evaluate the performance of the model [33].

**Table 2:** Public non-profit hospitals performance evaluation system.

Level 1	Weight ^a^	Level 2	Weight ^a^	Level 3,reference value	Weight ^b^	Comprehensive weight	Index attribute
Input	0.2	Human Resources	0.4	Percentage of health technicians (%), ≥75%	0.46	0.0365	+
				Doctors-nurses ratio, 1:2	0.54	0.0435	0
		Equipment and facilities	0.6	Beds-nurses ratio, 1:0.4	0.39	0.0471	0
				Percentage of fixed assets in total assets (%)	0.36	0.0437	+
				Average number of open beds	0.24	0.0293	+
Process	0.15	Nursing Management	0.3	The percentage of appropriate written nursing documents (%)	0.54	0.0242	+
				Percentage of passing student in nurses’ training (%)	0.46	0.0208	+
		Physician management	0.5	Percentage of passing student in doctors’ training (%)	0.25	0.0189	+
				Percentage of class A medical records in all medical records (%), ≥95%	0.26	0.0193	+
				The percentage of appropriate prescriptions (%)	0.22	0.0162	+
				Percentage of antibacterial prescription (%), 30–45%	0.27	0.0205	0
		Medical technology Management	0.2	Rate of CT inspection (%), ≥70%	0.13	0.0039	+
				Rate of MRI inspection (%), ≥70%	0.17	0.005	+
				Rate of X-ray inspection (%), ≥70%	0.17	0.0051	+
				Clinical chemistry laboratory scoring	0.18	0.0054	+
				Hematology laboratory scoring	0.11	0.0034	+
				Immunology laboratory scoring	0.12	0.0037	+
				bacteriological laboratory scoring	0.12	0.0035	+
Output	0.45	Quality	0.4	Therapeutic response rate (%)	0.13	0.0234	+
				Proportion of inpatients diagnosed within 3 days (%)	0.15	0.0273	+
				Mortality (%)	0.19	0.0349	-
				Proportion of nurses with basic qualification (%), ≥90%	0.12	0.0221	+
				Success rate of rescue (%)	0.13	0.0234	
				Incidence of nosocomial infection (%), ≤10%	0.14	0.0251	-
				Percentage of agreement between admission and discharge diagnoses (%), ≥95%	0.13	0.0239	+
		Efficiency	0.25	Medical institution bed utilization ratio (%), ≥90%	0.19	0.0214	+
				Medical institution bed turnover ratio, ≥19 times per year	0.29	0.0327	+
				Daily number of clinic patients for each doctor	0.19	0.0213	0
				Daily number of hospitalization bed-days for each doctor	0.16	0.0183	0
				Average number of days in hospital, ≤15 days	0.17	0.0187	-
		Cost control	0.15	Average outpatient expenditures (Yuan)	0.26	0.0176	-
				Average hospitalization expenditures (Yuan)	0.25	0.0171	-
				Average expenditures per bed per day (Yuan)	0.23	0.0155	-
				Percentage of medicine income of the total income, ≤45%	0.26	0.0173	-
		Financial balances	0.2	The asset-liability ratio (%)	0.18	0.0165	-
				Percentage of expenditures in service revenue (Yuan)	0.35	0.0314	-
				Income generated by each staff member (Yuan)	0.2	0.0181	+
				Medical income per 100 Yuan of fixed assets (Yuan)	0.27	0.024	+
Effect	0.2	Satisfaction	0.35	Patient satisfaction (%)	1	0.07	+
		Medical Safety	0.65	Compensation as a percentage of total income (%)	0.43	0.0554	-
				Medical accident rate per 10,000 inpatients	0.57	0.0746	-

^a^ Analytic hierarchy process (AHP) was used to determine the weights of level 1and 2 indicators; ^b^ The entropy weight method was used to determine the weights of level 3 indicators; The reference values were from Hospital management evaluation guidelines; +: Higher indicator values indicate better performance, -: Smaller indicator values indicate better performance, 0: values in one interval indicate better performance.

## 3. Results

The input-output model was used as the framework for establishing the performance evaluation system for public non-profit hospitals. Experts’ suggestion and criteria from the literature were used to select indicators. The weights of the indicators were determined by AHP and the entropy weight method (as shown in Table 2).

Based on the public non-profit hospitals performance evaluation system, the TOPSIS evaluation method was adopted to calculate the relative scores for the evaluation standards, as shown in Table 3.

**Table 3:** The weighted TOPSIS results for 14 level 3 hospitals in the first half of 2012.

Hospital code	The 1st half of 2012	
	*C_i_*	Rank
H1	0.6436	2
H2	0.6752	1
H3	0.6369	3
H4	0.6257	4
H5	0.4945	9
H6	0.4261	14
H7	0.5101	7
H8	0.4923	10
H9	0.4913	11
H10	0.4804	12
H11	0.4996	8
H12	0.5551	6
H13	0.4621	13
H14	0.5855	5

*C_i_* is relative approach degree in the TOPSIS method; The higher the value of *C_i_*, the better the rank.

The 14 public non-profit hospitals we chose are almost the same level (level 3, class A) and scale. The value of *C_i_* (relative approach degree) indicated the performance of the hospital. The higher the value of *C_i_*, the better the performance was. Thus, *C_i_* was the output variable for training and testing BP neural network.

**Figure 2:**
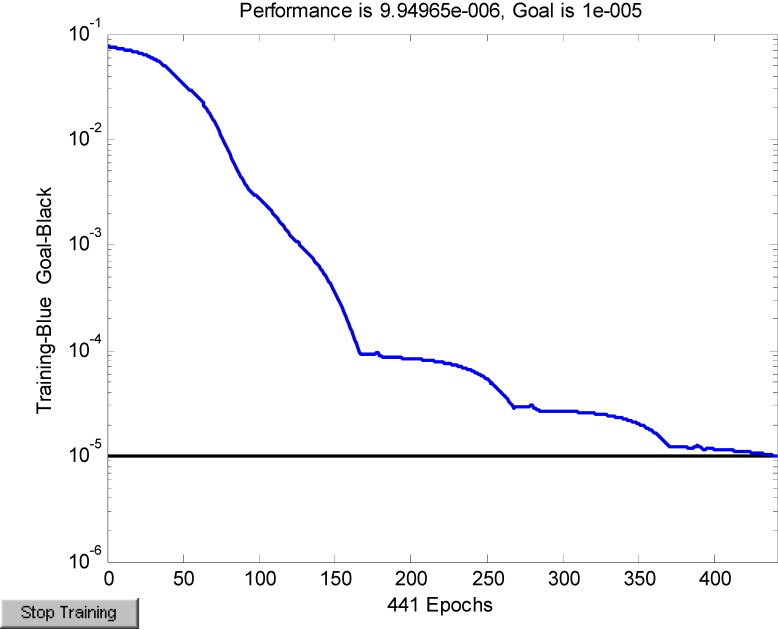
Training convergence curve.

Because the sample was small (14 hospitals), LOOCV was used to train and test the BP neural network to derive reliable results and increase the generation ability. The simulation of the training error is shown in Figure 2. The MSE value decreased as the number of iterative steps increased. Beginning with the 441th iterative step, the MSE was 9.95e−6, which was smaller than target error goal MSE (10^−5^). Consequently, the training was finished. The MATLAB software function “cputime” was adopted to record the computational cost of the model training, and the cpu time used to run the program is 60.35 s.

The statistical indicators of network performance are shown in Table 4. The network was trained by using LOOCV, and after 14 experiments, the average RMSE was 0.0392. The coefficient of determination (*R*^2^) was 0.9903. The closer that the value of *R*^2^ is to 1, the better the network performance is [34].

**Table 4:** The statistical indicators of net performance.

Model	Public Hospital Performance
Structure	41-10-1
RMSE	0.0392
*R*^2^	0.9903

The error analyses of partial least-squares regression are shown in Table 5. The coefficient of determination (*R*^2^) of PLSR model was 0.7731. Obviously, the value of *R*^2^ is smaller than BP neural network model. Thus, BP neural network provided the better results in performance evaluation for public non-profit hospital.

**Table 5:** The error analyses of partial least-squares regression.

Hospital code	Observed value	Prediction value	Absolute error	Relative error (%)
H1	0.6436	0.6377	0.0059	0.92
H2	0.6752	0.6242	0.0510	7.55
H3	0.6369	0.6225	0.0144	2.27
H4	0.6257	0.5879	0.0378	6.03
H5	0.4945	0.5405	−0.0460	9.31
H6	0.4261	0.4526	−0.0265	6.22
H7	0.5101	0.5374	−0.0273	5.35
H8	0.4923	0.5429	−0.0506	10.29
H9	0.4913	0.4674	0.0239	4.87
H10	0.4804	0.5051	−0.0247	5.15
H11	0.4996	0.5619	−0.0623	12.46
H12	0.5551	0.5217	0.0334	6.01
H13	0.4621	0.4817	−0.0196	4.24
H14	0.5855	0.6410	−0.0555	9.47

## 4. Conclusions

The performance evaluation system for public non-profit hospitals covers almost all aspects of quality for public non-profit hospitals. The system could reflect the guidance of public non-profit hospitals reform. The indicator weights were scientific and reasonable, based on both the objective and the subjective points of view. Developing the indicator weights using both the subjective (experts scoring and AHP) and objective (entropy method) methods ensured that both experience and objectivity were considered.

The integrity and the accuracy of the data were also important and, may have influenced the results. A small mistake could potentially lead to inaccurate estimates of the hospital's performance. HMSIQCC has a special quality control measure for the integrity and accuracy of the management data for public non-profit hospitals. All of the data used in this study were verified.

A relatively stable BP neural network model was obtained using the Leave-one-out Cross-Validation method and adjusting the network parameters. According to the results, the structure of the BP neural network was 41-10-1, *R*^2^ was 0.9903 and RMSE was 0.0392. Compared with PLSR model, the value of *R*^2^ (*R*^2^ = 0.9903) for BP neural network is larger than PLSR model (*R*^2^ = 0.7731). Thus, the proposed model could be used for public non-profit hospital performance evaluations.

The new health reform policies in our country state that three key problems should be considered: accessibility, equity and price. Accessibility refers to basic medical institutions. Equity refers to the efforts to narrow the gap between urban and rural areas and between regions, and the price is a consideration so that people can afford to consult a doctor or purchase medicines when they get sick. Thus, a performance evaluation system for public non-profit hospitals should have certain characteristics (*i.e.*, the weights of the social benefit index in the evaluation system should be increased). Consequently, the commonweal goal of public non-profit hospitals can be reflected fully.

One of the purposes of this evaluation is to help the government understand the performance of public non-profit hospitals and to aid in decisions related to the development and reform of public non-profit hospitals; another goal is to help hospitals improve their performance. Whether or not performance evaluation improves performance depends on whether and how the evaluation results are applied [35]. First, the evaluation results should be used appropriately. Each public non-profit hospital will find the problems or weak links that may influence the evaluation results. Then, they may resolve or improve them, but they may accomplish these results falsifying data or accepting only low-risk patients. Second, the evaluation system and methodology should be same, so that the performance evaluation results from different hospitals will be comparable. Third, the performance of hospitals at different levels and in different categories should be evaluated individually. 
